# Supplementary material for: Analytical performance evaluation of a multiplex real-time RT-PCR kit for simultaneous detection of SARS-CoV-2, influenza A/B, and RSV
Source: PeerJ. 2025 Jul 30;13:e19693. doi: 10.7717/peerj.19693 (PMC12317686; doi:10.7717/peerj.19693)
Supplement: Supplemental Information 2 [file peerj-13-19693-s002.doc]

STROBE Statement—Checklist of items that should be included in reports of ***cross-sectional studies***

|  | Item No | Recommendation |
| --- | --- | --- |
| **Title and abstract** | 1 | (*a*) Indicate the study’s design with a commonly used term in the title or the abstract addressed in page 2 |
|  |  | (*b*) Provide in the abstract an informative and balanced summary of what was done and what was found addressed in page 2 |
| Introduction | | |
| Background/rationale | 2 | Explain the scientific background and rationale for the investigation being reported addressed in page 3-4 |
| Objectives | 3 | State specific objectives, including any prespecified hypotheses addressed in page 5-6 |
| Methods | | |
| Study design | 4 | Present key elements of study design early in the paper addressed in page 6 |
| Setting | 5 | Describe the setting, locations, and relevant dates, including periods of recruitment, exposure, follow-up, and data collection addressed in page 2 |
| Participants | 6 | (*a*) Give the eligibility criteria, and the sources and methods of selection of participants addressed in page 2 |
| Variables | 7 | Clearly define all outcomes, exposures, predictors, potential confounders, and effect modifiers. Give diagnostic criteria, if applicable |
| Data sources/ measurement | 8* | For each variable of interest, give sources of data and details of methods of assessment (measurement). Describe comparability of assessment methods if there is more than one group |
| Bias | 9 | Describe any efforts to address potential sources of bias addressed in page 18 |
| Study size | 10 | Explain how the study size was arrived at N/A |
| Quantitative variables | 11 | Explain how quantitative variables were handled in the analyses. If applicable, describe which groupings were chosen and why addressed in page 6 |
| Statistical methods | 12 | (*a*) Describe all statistical methods, including those used to control for confounding addressed in page 13 |
|  |  | (*b*) Describe any methods used to examine subgroups and interactions addressed in page 8-11 |
|  |  | (*c*) Explain how missing data were addressed N/A |
|  |  | (*d*) If applicable, describe analytical methods taking account of sampling strategy addressed in page 11 |
|  |  | (*e*) Describe any sensitivity analyses addressed in page 11 |
| Results | | |
| Participants | 13* | (a) Report numbers of individuals at each stage of study—eg numbers potentially eligible, examined for eligibility, confirmed eligible, included in the study, completing follow-up, and analysed addressed in page 7 |
|  |  | (b) Give reasons for non-participation at each stage N/A |
|  |  | (c) Consider use of a flow diagram addressed in page 7 |
| Descriptive data | 14* | (a) Give characteristics of study participants (eg demographic, clinical, social) and information on exposures and potential confounders |
|  |  | (b) Indicate number of participants with missing data for each variable of interest |
| Outcome data | 15* | Report numbers of outcome events or summary measures addressed in page 15-16 |
| Main results | 16 | (*a*) Give unadjusted estimates and, if applicable, confounder-adjusted estimates and their precision (eg, 95% confidence interval). Make clear which confounders were adjusted for and why they were included addressed in page 15-16 |
|  |  | (*b*) Report category boundaries when continuous variables were categorized N/A |
|  |  | (*c*) If relevant, consider translating estimates of relative risk into absolute risk for a meaningful time period N/A |
| Other analyses | 17 | Report other analyses done—eg analyses of subgroups and interactions, and sensitivity analyses addressed in page 15-16 |
| Discussion | | |
| Key results | 18 | Summarise key results with reference to study objectives addressed in page 16 |
| Limitations | 19 | Discuss limitations of the study, taking into account sources of potential bias or imprecision. Discuss both direction and magnitude of any potential bias addressed in page 18 |
| Interpretation | 20 | Give a cautious overall interpretation of results considering objectives, limitations, multiplicity of analyses, results from similar studies, and other relevant evidence addressed in page 16 |
| Generalisability | 21 | Discuss the generalisability (external validity) of the study results addressed in page 17 |
| Other information | | |
| Funding | 22 | Give the source of funding and the role of the funders for the present study and, if applicable, for the original study on which the present article is based N/A |

*Give information separately for exposed and unexposed groups.

**Note:** An Explanation and Elaboration article discusses each checklist item and gives methodological background and published examples of transparent reporting. The STROBE checklist is best used in conjunction with this article (freely available on the Web sites of PLoS Medicine at http://www.plosmedicine.org/, Annals of Internal Medicine at http://www.annals.org/, and Epidemiology at http://www.epidem.com/). Information on the STROBE Initiative is available at www.strobe-statement.org.
